# Recurring Bleeding Events Requiring Red Blood Cell Transfusion After Left Atrial Appendage Closure Are Associated with Increased Mortality

**DOI:** 10.3390/jcm15072626

**Published:** 2026-03-30

**Authors:** Manuella Bogdan, Balázs Polgár, Előd János Zsigmond, Jusztina Bencze, Kamilla Luca Dávid, Zalán Gulyás, Nikolett Vigh, Róbert Gábor Kiss, Emese Tóth-Zsámboki, Gábor Zoltán Duray

**Affiliations:** 1 Department of Cardiology, Central Hospital of Northern Pest—Military Hospital, 1134 Budapest, Hungary; manuella.bogdan@gmail.com (M.B.);; 2 Cardiovascular Medicine and Research Division, Semmelweis University Doctoral School, 1085 Budapest, Hungary; kamillalucadavid@gmail.com; 3 Heart and Vascular Center, Semmelweis University, 1085 Budapest, Hungary

**Keywords:** atrial fibrillation, left atrial appendage closure, watchman device, stroke prevention, oral anticoagulation contraindication, bleeding risk

## Abstract

**Background:** Left atrial appendage closure (LAAC) is an established alternative to oral anticoagulation for stroke prevention in patients with nonvalvular atrial fibrillation who are at high risk of thromboembolic events or bleeding complications. **Methods:** In this single-center retrospective study, we analyzed 70 consecutive patients who underwent successful LAAC with the Watchman™ device between 2012 and 2024. Acute procedural outcomes, long-term thromboembolic and bleeding events, transfusion requirements and mortality were evaluated. Mean follow-up duration was 1210 days. **Results:** Procedural success was achieved in 98.6% of cases with a low periprocedural complication rate. Ischemic stroke/transient ischemic attack occurred in 2.8% of patients; no hemorrhagic strokes or stroke-related deaths were observed. LAAC resulted in a significant reduction in both the number (144 vs. 56 events; 2.36 vs. 1.55 events per patient, *p* < 0.05) and severity of bleeding events. Nonetheless, 42.9% of patients required bleeding-related hospitalization after implantation, predominantly within the first 6 months during dual antiplatelet therapy. Overall mortality was 40% with a 12% yearly mortality rate; heart failure and infections were leading causes of death. Pre- and postprocedural transfusion requirements were independently associated with a six-fold increase in mortality risk (HR = 5.97). Conventional risk scores (CHA_2_DS_2_-VASc, HAS-BLED) failed to predict transfusion needs; atrial enlargement, right ventricular dysfunction, smoking and alcohol consumption were associated with higher risk. **Conclusions:** LAAC is a safe and effective alternative to long-term anticoagulation, significantly reducing bleeding burden without increasing thromboembolic mortality. Persistent postprocedural bleeding remains a major determinant of long-term prognosis, underscoring the need for individualized, multidisciplinary post-implant management.

## 1. Introduction

Atrial fibrillation (AF) is the most common sustained arrhythmia and a leading cardiac cause of ischemic stroke [1]. Its prevalence continues to rise with the aging population. The standard of care for stroke prevention in patients with AF is oral anticoagulation (OAC), either with vitamin K antagonists (VKAs) or direct oral anticoagulants (DOACs). However, several clinical situations may arise in which long-term OAC cannot be safely administered or maintained despite a clear indication for stroke prevention. Severe renal or hepatic dysfunction, thrombocytopenia, documented intolerance with hypersensitivity reactions, and clinically significant drug interactions may complicate treatment. Reduced efficacy of anticoagulation had previously been suggested in patients with severe obesity; however, recent meta-analyses have not confirmed this concern [2]. In addition, practical barriers to safe long-term therapy—such as cognitive impairment, poor adherence, or difficulties with regular monitoring and medication management—may further limit OAC use. These challenges frequently coexist with comorbidities that substantially increase hemorrhagic risk, most commonly advanced age and frailty. While anticoagulant therapy is effective in preventing cardioembolic events, an increased risk of bleeding episodes is clearly present. The balance of bleeding and thrombotic risks should be considered in each patient individually. A substantial subset of patients remain unsuitable for sustained anticoagulant therapy, and in these individuals, closure of the left atrial appendage represents an important alternative strategy for stroke prevention.

The left atrial appendage (LAA), a remnant of the embryonic left atrium, is the primary site of thrombus formation in nonvalvular AF, accounting for over 90% of cases [3]. Left atrial appendage closure (LAAC) became a device-based alternative to OAC, particularly for patients at high risk of thromboembolic events and/or bleeding [4,5]. The 2024 European Society of Cardiology (ESC) guidelines for the management of AF recommend percutaneous LAA occlusion in patients with contraindications to long-term anticoagulation to reduce the risk of ischemic stroke and thromboembolism (Class IIb, Level C) [6]. Similarly, the 2023 ACC/AHA/ACCP/HRS guidelines upgraded the recommendation to Class IIa, reflecting growing support for this strategy in patients unsuitable for OAC [7]. Registry and large-scale clinical studies provide data for the efficacy of LAAC. However, its capability to reduce bleeding events is less extensively documented and studied.

The aim of our study was to evaluate the acute and long-term safety and efficacy of percutaneous LAA closure with the Watchman^TM^ device, with focus on a detailed analysis of bleeding complications. We compared outcomes between patients with recurrent thromboembolic events and with increased bleeding risk and sought to identify factors associated with mortality and major adverse and bleeding events following the procedure.

## 2. Methods

This retrospective, single-center study analyzed 70 consecutive patients who underwent successful left atrial appendage closure (LAAC) between 2012 and 2024 at our institution. The technical success rate of left atrial appendage closure with the Watchman occluder was high (98.6%). Seventy of the 71 initiated procedures were successfully completed. One patient underwent surgical left atrial appendage closure due to pericardial tamponade prior the LAAC implementation; consequently, this patient was excluded from the study.

All patients had nonvalvular atrial fibrillation (AF) and either a contraindication to anticoagulation due to serious bleeding and/or high bleeding risk, or experienced recurrent thromboembolic events despite treatment with vitamin K antagonists (VKAs) or direct oral anticoagulants (DOACs).

Clinical and procedural data were extracted from the hospital’s electronic medical records system (MedWorks) and the National eHealth Infrastructure (EESZT). Bleeding events were retrospectively independently adjudicated by two authors through systematic review of the above databases, discharge summaries and transfusion documentation. Written informed consent was obtained from all eligible participants. The study protocol was approved by the Institutional Ethics Committee (approval number 233-1/2023) and was conducted in accordance with the Declaration of Helsinki.

In patients without in-hospital mortality, the cause of death was classified as unknown. Available official death certificates, issued by hospitals, were independently reviewed by two authors for each individual case. Baseline clinical assessments included patient history, routine laboratory tests, chest X-ray, and transthoracic echocardiography (TTE), all performed prior to discharge from the hospital.

## 3. Procedure

All patients underwent left atrial appendage closure (LAAC) under conscious sedation, guided by transesophageal echocardiography (TOE). Intravenous unfractionated heparin was administered at a dose of 80–100 IU/kg following transseptal puncture to maintain adequate anticoagulation during the procedure. TOE was performed prior to the intervention to exclude the presence of intracardiac thrombus, particularly in the left atrial appendage.

Percutaneous access was obtained via the femoral vein, and a transseptal puncture was performed under TOE guidance to access the left atrium. A Watchman™ device (after 2022 Watchman FLX™, Boston Scientific Corporation, Marlborough, MA, USA) was deployed into the left atrial appendage under fluoroscopic and echocardiographic visualization, ensuring appropriate device positioning and seal. Device size was selected based on LAA measurements from intra-procedural imaging.

After satisfactory device deployment and confirmation of position, stability, and absence of peri-device leak, the delivery catheter was withdrawn and hemostasis was achieved at the vascular access site. Postprocedural transthoracic echocardiography was performed before discharge to assess pericardial effusion or other immediate complications.

The sheath type, implantation technique and overall procedural workflow was stable over time. Procedures were performed by the same operator team, namely 2 senior operating electrophysiologists.

Concerning standard postoperative anticoagulant and antiplatelet therapy—since patients were considered at high bleeding risk—dual antiplatelet therapy (DAPT) was prescribed for 6 months, parallel with OAC discontinuation after the procedure. An exception was made in the case of one patient with pulmonary embolism (persistent DOAC) and one patient with massive postprocedural gastrointestinal bleeding (single antiplatelet therapy, SAPT). After 6 months, lifelong SAPT was suggested for all patients.

### Statistical Analysis

All statistical analyses were performed using Statistica 10 software (StatSoft Inc., Tulsa, OK, USA). The Shapiro–Wilk test was used to assess the normality of continuous variables. Data with a normal distribution are presented as mean ± standard error of the mean (SEM), while non-normally distributed data are reported as median and interquartile range (IQR). Most of the variables did not follow a normal distribution; therefore, non-parametric tests were employed. Differences between independent subgroups were analyzed using the Mann–Whitney U test, and repeated measures were compared using the Wilcoxon signed-rank test. Categorical variables were assessed with Fisher’s exact or—for paired categorical data—Mc Nemar test.

Survival analyses were conducted using Kaplan–Meier curves and survival tables, where survival curves were constructed with the date of LAAC implantation defined as time zero. Time-to-event was calculated from the implantation date to death from any cause or the last documented clinical contact. Patients who were alive at the end of follow-up were censored at the date of their last available clinical evaluation. Associations with comorbidities were evaluated using the Cox proportional hazards model and were demonstrated by Forest plots. ROC analysis and multivariate Cox analysis were performed to quantify the impact of postoperative transfusions on mortality.

## 4. Results

### 4.1. Patients Demographics

The study cohort consisted of 70 patients who underwent successful left atrial appendage closure with a Watchman occluder. Table 1 displays patient demographics with a balanced gender distribution (53% male, 47% female) and a median age of 75.5 years. Patients were divided into two subgroups based on indication of the procedure: recurrent stroke or thromboembolic events despite ongoing anticoagulant therapy (n = 8) and high bleeding risk or contraindication of anticoagulant therapy (n = 62). Hypertension was the most common comorbidity (93% overall), followed by hyperlipidemia (64%) and chronic kidney disease (61%).

Risk assessment was performed using the CHA_2_DS_2_-VASc, CHA_2_DS_2_-VA and HAS-BLED scores to quantify thromboembolic and bleeding risks. The median CHA_2_DS_2_-VASc score was five (IQR 4–6) in the overall cohort, reflecting a high baseline risk for stroke. This was even slightly higher in the recurrent stroke/thromboembolic subgroup (median six (IQR 5–6.5)). HAS-BLED score was elevated across all groups with a median of four (IQR 4–5).

Echocardiography indicated structural abnormality, namely significantly larger right ventricle, left and right atrial diameters in patients with high bleeding risk. Despite recurrent thromboembolic events, the above parameters were normal in the other patient subgroup. Laboratory parameters and medical therapy at baseline and discharge are shown in Supplement Tables S1 and S2, respectively.

The demographics of survivor (n = 42) and deceased (n = 28) patient populations are indicated in Supplement Table S3. Deceased patients had a greater burden of cardiovascular and metabolic comorbidities, including obesity, diabetes, hyperlipidemia, coronary artery disease (CAD), and a history of myocardial infarction (AMI) or stroke, indicating an elevated risk profile for both metabolic and thromboembolic complications. They also had significantly higher CHA_2_DS_2_-VASc and CHA_2_DS_2_-VA scores, supporting their ability to predict mortality.

### 4.2. Procedure-Related Acute and Long-Term Complications

Complications were infrequent and occurred as isolated events. Pericardial effusion and tamponade were the most common complications in 1.4% and 1.4% of patients, respectively. Other events included gastrointestinal bleeding (1.4%), vascular complications (2.8%), esophageal injury (1.4%) and thrombus formation upon catheter insertion (1.4%). Importantly, there were no procedure-related deaths or periprocedural ischemic strokes.

Long-term procedural complications were rare (1.4%); one patient, later diagnosed with Factor V Leiden mutation, developed a device-related thrombus and was managed conservatively without clinical deterioration or thromboembolic event. Importantly, no significant peri-device leak was reported during the 3-month follow-up TOE (Supplement Table S4).

### 4.3. Anticoagulant and Antiplatelet Therapy

Table 2 indicates antiplatelet and anticoagulant therapy on admission and at discharge. Half of the population with high bleeding risk received LMWH therapy on admission. As described above (Methods), after the procedure, standard dual antiplatelet therapy was applied in 88.6% of the total population and LMWH was fully discontinued. DOAC or VKA therapy were maintained in 50% of the patients with recurrent stroke/thromboembolic events. In our high-risk elderly cohort undergoing LAAC, OAC was universally discontinued due to bleeding risk (DOAC and VKA were withdrawn in 96.8% of patients with high bleeding risk).

Standard dual antiplatelet therapy (clopidogrel 75 mg + ASA 100 mg daily) was administered for 6 months, followed by lifelong single antiplatelet therapy, as per the manufacturer-approved Watchman regimen. In two patients with major bleeding, therapy was reduced to a single antiplatelet (ASA in one, clopidogrel in one), without device-related thrombosis.

OAC was re-initiated in two patients experiencing intracardiac thrombus and pulmonary embolism. About 30% of patients were followed at our tertiary center, with the remainder at regional outpatient clinics. After 6 months, most continued SAPT; OAC was altogether re-initiated in six patients (four with high thromboembolic risk) combined with clopidogrel.

### 4.4. Major Adverse Events During Follow-Up: Stroke, Cardiovascular Events, New-Onset Malignancy and Mortality

#### 4.4.1. Stroke

Rates of ischemic stroke (1.4%) and transient ischemic attack (1.4%) were both low, with no reported cases of hemorrhagic stroke (Table 3).

#### 4.4.2. Cardiovascular Events

During long-term follow-up (average 1210 days), the incidence of coronary revascularization was 10%, predominantly in the high-bleeding-risk group (9.7%, Table 3). Acute myocardial infarction (AMI) occurred in a single patient (1.4%). Heart failure requiring hospitalization was more common, affecting 17.1% of the cohort. Pacemaker implantation occurred in 10% of patients.

#### 4.4.3. New-Onset Malignancy

New-onset malignancy occurred in patients with high bleeding risk during follow-up (12.9%, Table 3). Median time until diagnosis of malignancy was 442.5 days after the procedure.

#### 4.4.4. Mortality Rate, Cause of Death and Clinical Parameters Affecting Mortality

**Figure 1:**
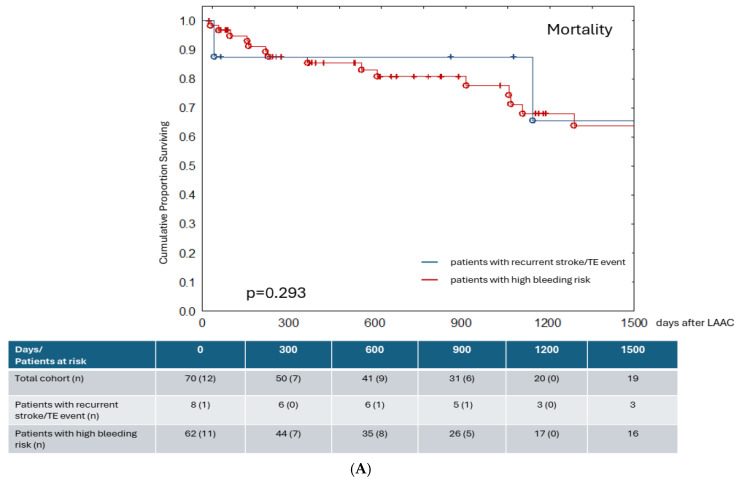

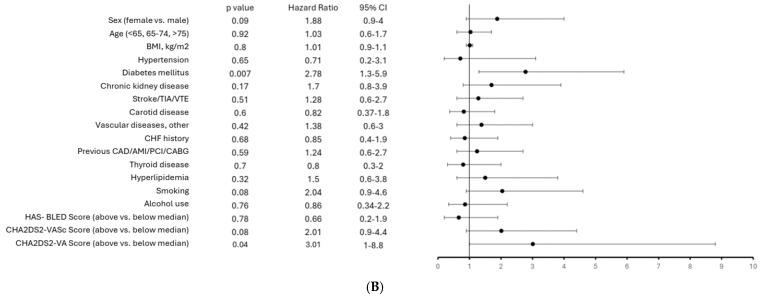
(**A**) Kaplan–Meier curve with log-rank test and survival table for overall survival of subgroups of patients with recurrent stroke/TE event (blue line) and high bleeding risk (red line) at 1500 days follow-up after LAAC. No significant statistical difference was found between subgroups in respect to survival (*p* = 0.293, log-rank test). (**B**) Forest plot for Cox proportional hazard model for clinical and demographic parameters regarding mortality. Hazard ratio and 95% confidence intervals are provided for each variable for the event of death in total cohort. Abbreviations: AMI: acute myocardial infarction, BMI: body mass index, CAD: coronary artery disease, CABG: coronary artery bypass grafting, CHF: chronic heart failure, PCI: percutaneous coronary intervention, TIA: transient ischemic attack, VTE: venous thromboembolism.

During follow-up, total mortality was 40%, with 12.9% in the first year (Table 3). We determined long-term survival outcomes of patients with recurrent stroke/thromboembolic events and high bleeding risk following LAAC (Figure 1A); long-term survival did not differ significantly between these patient subgroups (*p* = 0.293, log-rank test).

Leading causes of death were heart failure (affecting only the high-bleeding-risk group) and infectious diseases, particularly septicemia and COVID-19 pneumonia. It is important to note that stroke and hemorrhagic shock—as efficacy endpoints—were not reported as a cause of death, suggesting that LAAC has contributed to effective stroke and bleeding prevention in this high-risk population.

The forest plot for the Cox proportional hazard model (Figure 1B) suggests that diabetes mellitus and higher CHA_2_DS_2_-VA scores are statistically significant predictors of death in this cohort. Other comorbidities and risk scores, including HAS-BLED, did not show significant associations. Several factors (e.g., hyperlipidemia, smoking, previous stroke/TIA/VTE) show trends toward increased risk but are not statistically significant and may require a larger sample size for confirmation.

### 4.5. Analysis of Bleeding Events Before and After LAAC Implantation

#### 4.5.1. Definition and Classification of Pre- and Postoperative Bleeding Events

The majority of patients were referred for the LAAC procedure due to bleeding episodes or high bleeding risk for anticoagulant therapy. We did not observe bleeding episodes in the recurrent thromboembolic/stroke subgroup. Therefore, we analyzed bleeding-related outcomes and clinical events in high-bleeding-risk patients.

Pre- and postoperative bleeding events were individually analyzed by using multiple standard classification systems concerning severity and origin of hemorrhage (BARC, ISTH and TIMI). Similarly, hospitalization and transfusion requirements were recorded for each bleeding event within the high-bleeding-risk patient subgroup.

#### 4.5.2. Reduced Bleeding Frequency, Severity, Bleeding-Related Hospitalization and Transfusion Were Observed After LAAC

Most importantly, the number of registered bleeding events and patients experiencing bleeding episodes were significantly reduced after LAAC implantation (144 events/61 patients vs. 56 events/30 patients; McNemar test, * *p* < 0.001), indicating that withdrawal of anticoagulant therapy significantly reduced these events (Table 4). However, it is important to emphasize that bleeding episodes were not completely eliminated; a substantial number of patients suffered recurrent bleeding events, bleeding-related rehospitalization and required transfusion after the procedure (Table 4).

**Figure 2:**
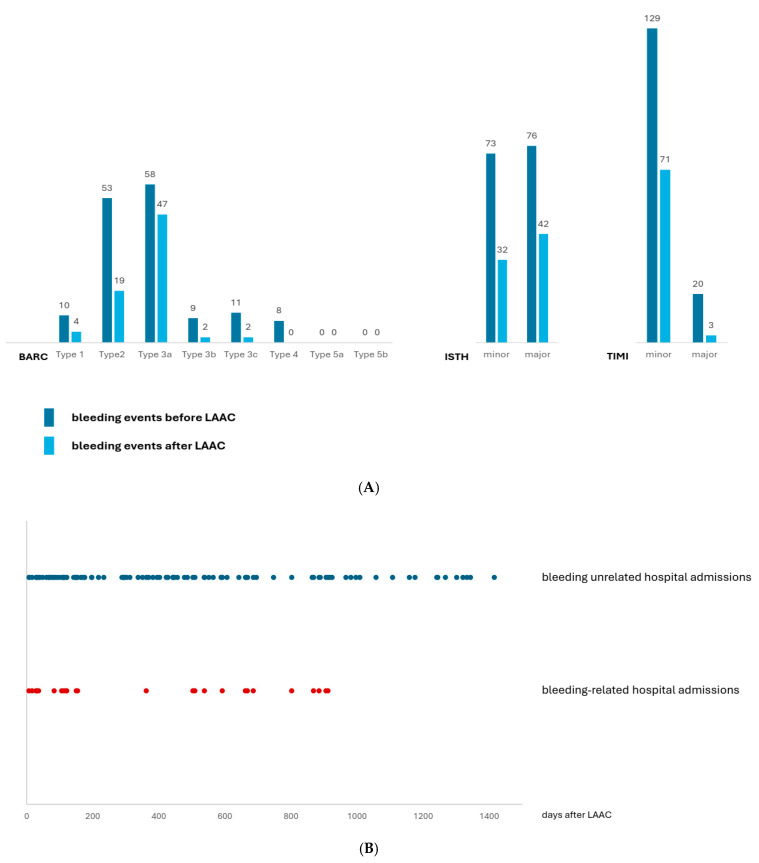

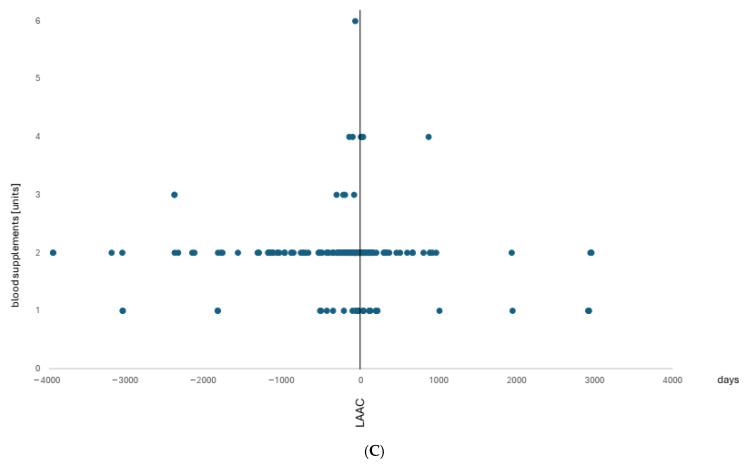
(**A**). Number of bleeding events before and after LAAC in the total population. Every bleeding event was classified according to BARC, ISTH and TIMI scores. Bleeding events were significantly reduced after LAAC implantation in each bleeding classification system. (**B**). Bleeding-unrelated and bleeding-related hospital admissions after the procedure in total population. Each dot represents one hospitalization episode. (**C**). Administered red blood cell supplements before and after LAAC in patients with high bleeding risk. Each dot represents an individual transfusion procedure with the administered red blood cell supplements indicated in units on the y scale.

**Figure 3:**
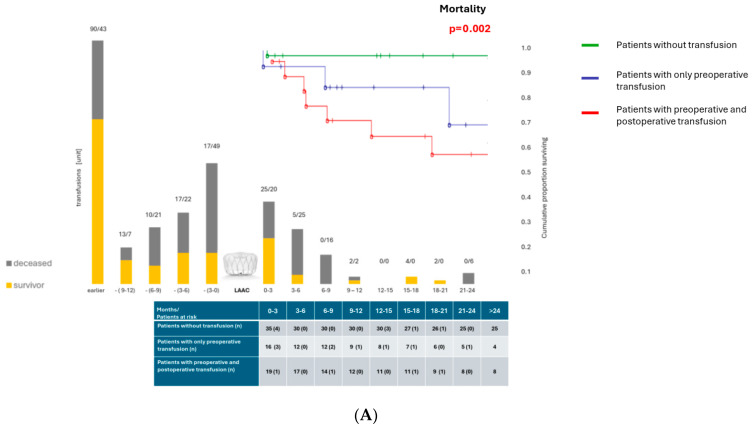

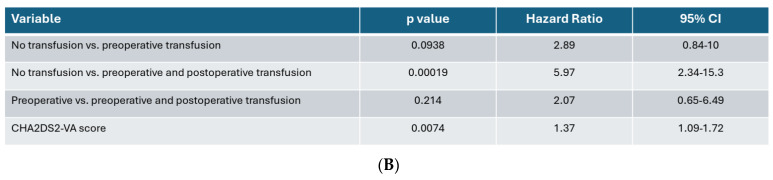
(**A**) Administered red blood cell supplements before and after LAAC in survivor and deceased subgroups within the total population. Number of transfused red blood cell supplements in deceased (gray) and survivor patients (yellow) are indicated on top of each column, representing 3-month periods. Parallel Kaplan–Meier survival curve and survival table of non-transfused (green line), only preoperative-transfused (blue line) and both preoperative- and postoperative-transfused patients (red line) indicating significantly increased mortality in transfused patients (log-rank test, *p* = 0.002). (**B**) Mortality in the context of pre- and postoperative bleeding events requiring transfusions and CHA2DS2-VA score. The table reports the hazard ratio and the 95% confidence intervals of the hazard ratio for each variable included in the multivariate Cox proportional hazard model analysis for the event of death in total cohort.

The majority of postprocedural bleeding episodes were observed within the first 6 months following LAAC implantation (24 events out of 56 in total), temporally corresponding to the dual antiplatelet therapy phase. Postprocedural transfusions and hospitalizations (Figure 2B,C and Figure 3A, Table 5) all follow the same pattern.

Concerning the severity of bleeding episodes, major bleeding (ISTH and TIMI) showed more than 50% reduction and fatal bleeding (BARC types 5a and 5b) was absent after the procedure (Figure 2A). The TIMI minor bleeding category was the most common, showing a notable drop as well (Figure 2A). Gastrointestinal bleeding remained the most common etiology in both pre- and postoperative periods, though its frequency declined by more than half (39 vs. 16 total event). Intracranial hemorrhage, present in eight cases preoperatively and representing 13% of total bleeding episodes, was entirely absent after the procedure. Other bleeding types, including hematuria, epistaxis, and rare combinations such as hemarthrosis or scrotal bleeding, were only observed in isolated postoperative cases. Procedure-related bleeding complications included one case of pericardial tamponade and two cases of access site hematoma (Table 4).

Bleeding events frequently required hospital admissions following LAAC; bleeding-related hospitalization (n = 34) was reduced but not abolished (Figure 2B, Table 4). After LAAC implantation, persistent bleeding risk was likely to be related to the applied DAPT, since the majority of post-LAAC hospitalization due to bleeding episodes were observed within the first six months of follow-up (Figure 2B). Unexpectedly, bleeding-unrelated admissions occur in an even larger number, continuously across the entire follow-up period (Figure 2B, Table 4).

In most cases, during bleeding-related hospitalizations, patients required red blood cell transfusions. The scatter plot presents administered red blood cell supplements (in units) before and after the LAAC procedure (Figure 2C). Prior to the procedure, transfusions were frequent and scattered across several years since bleeding problems were usually present for a prolonged period. After the procedure, the frequency and intensity of transfusions drop substantially, supporting procedural efficacy in bleeding reduction. The number of transfused patients was 19 vs. 33 pre-LAAC; the total number of administered red blood cell supplements was 134 vs. 289 units pre-LAAC, consistently indicating successful reduction of overall bleeding burden (Table 4). Importantly, at the individual level, the number of administered red blood cell transfusions/patient was also reduced (7.1 U/pt vs. pre-LAAC 8.8 U/pt, Table 4). Still, even in the long term, the need for blood supplement administration is not fully eliminated.

Deceased high-bleeding-risk patients experienced more postoperative bleeding events (34 vs. 22), bleeding-related hospitalizations (23 vs. 11), and required more blood transfusions (7.8 vs. 6 units/patient) than survivors.

#### 4.5.3. Impact of Bleeding on Mortality: Data from Patients with Pre-/Postprocedural Transfusions

Within the high-bleeding-risk cohort, 19 out of 62 patients (30,67%) required postoperative transfusion. Patients receiving red blood cell transfusion following LAAC exhibited significantly higher mortality compared to those without transfusion: 63.2% vs. 27.9% overall mortality, and 31.6% vs. 4.6% one-year mortality, respectively (Table 5).

In order to further evaluate the effect of pre- and postoperative transfusion on mortality, we analyzed data via Kaplan–Meier survival analysis (Figure 3A) on the following subgroups: patients without transfusion (n = 35), patients with only preoperative (n = 33), and patients with pre- and postoperative (n = 19) transfusion requirements. In order to reduce the number of covariates and the event-per-variable ratio during subsequent statistical analyses, two patients, requiring transfusion only after the procedure, were included in the last category. We found significant difference in long-term outcomes among these subgroups, with patients receiving pre- and postoperative transfusion being at highest risk (*p* = 0.02, log-rank test). The survival curves diverged early and continued to separate throughout the follow-up period. At approximately 1200 days post-procedure, survival remained above 85% in the non-transfusion group, while it declined to around 50% in the group with pre- and postoperative transfusion.

To address the potential risk of immortal-time bias and reverse causation, we performed landmark survival analyses at 45 days and 90 days after LAAC implantation. Kaplan–Meier survival curves consistently and significantly were separated, indicating reduced long-term survival among patients requiring postprocedural transfusion. This association remained significant both at the 45-day (log-rank *p* < 0.0001) and at the 90-day landmark (log-rank *p* = 0.002).

To analyze the effect of pre- and postoperative transfusion need, we used the multivariate Cox proportional hazard model (Figure 3B). As demonstrated in the table, patients requiring both pre- and postoperative transfusions have a significantly higher risk of mortality compared to those without transfusions (HR = 5.97, *p* = 0.00019, highly significant). Even compared with only preoperative transfusion, adding postoperative transfusion increases risk (HR 2.07), but did not reach statistical significance (*p* = 0.21). CHA_2_DS_2_-VA score was shown to be a significant independent predictor of mortality; our data confirm this observation (HR = 1.37, *p* = 0.007). Preoperative transfusion alone showed a trend toward increased mortality but was not statistically significant (*p* = 0.09).

Mortality in patients receiving 2, 2–10, or >10 red blood cell units was 62.5%, 60.0%, and 66.7%, respectively. ROC curve analysis, performed to evaluate the impact of bleeding severity, demonstrated that transfusion of ≥2 units was already independently associated with increased mortality, without evidence of a dose–response relationship beyond this threshold.

Because of the concern of heterogeneity, we performed a calendar-era sensitivity analysis by dividing the cohort into three prespecified periods: 2012–2016, 2017–2020, and 2021–2024. The basic event rates showed a marked decline in mortality over time, whereas transfusion rates remained relatively similar across eras. Mortality was 78.8% in 2012–2016, 55.6% in 2017–2020, and 19.1% in 2021–2024. Transfusion occurred in 26.3%, 22.2% and in 28.6% of patients, respectively.

To explore whether implantation year influenced the association between transfusion timing and mortality, we performed Cox regression analysis including year of LAAC as a covariate. In this model, year of LAAC was not associated with mortality (HR 1.02, 95% CI 0.88–1.17, *p* = 0.842), whereas postprocedural transfusion remained strongly associated with mortality compared with both no transfusion (HR 13.78, 95% CI 4.91–38.66, *p* < 0.001) and preprocedural transfusion only (HR 7.68, 95% CI 2.50–23.59, *p* < 0.001). CHA_2_DS_2_-VA score also remained significant (HR 1.43, 95% CI 1.14–1.79, *p* = 0.002).

#### 4.5.4. Analysis of the Residual Bleeding Risk After LAAC: Data from Patients Requiring Recurrent Transfusion After LAAC

The highest mortality risk was observed in patients demanding both rehospitalization and red blood cell transfusion after LAAC implantation (n = 19, 31.6% mortality in first year, Table 5). In order to aid clinical decision-making and identify these vulnerable patients before the LAAC procedure, we analyzed the demographics, bleeding/transfusion characteristics and mortality data of this subgroup (Table 5).

#### 4.5.5. Demographics

Prevalence of diabetes, chronic kidney disease, CAD, previous AMI and CABG was higher in patients with transfusion requirements. A higher rate of alcohol consumption and smoking were documented in this subgroup as well. Echocardiography showed enlarged right ventricle with reduced longitudinal systolic function and larger atrii (Table 5).

#### 4.5.6. Bleeding Characteristics

Characteristics of repetitive bleeding events after LAAC were similar to the total patient cohort; a specific condition explaining prolonged bleeding tendency was not found in these patients (Table 4).

#### 4.5.7. Factors Related to Postoperative Transfusion Need

**Figure 4:**
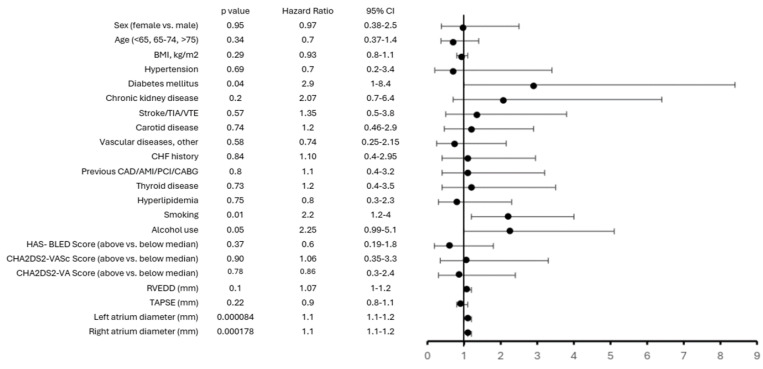
Forest plot for Cox proportional hazard model for clinical and demographic parameters regarding postoperative transfusion need. The figure provides a forest plot reporting hazard ratio and 95% confidence intervals of the hazard ratio for each variable for the event of transfusion in a subgroup of patients with high bleeding risk.

Interestingly, Cox regression highlights smoking, alcohol use, and left and right atrial enlargement as significant predictors of transfusion requirement after LAAC in patients with high bleeding risk. Other clinical comorbidities show trends but do not reach statistical significance (Figure 4).

Unexpectedly, CHA_2_DS_2_-VASc, CHA_2_DS_2_-VA and HAS-BLED scores (already all being in a high range) did not differ between the two subgroups (CHA_2_DS_2_-VASc *p* = 0.6, CHA_2_DS_2_-VA *p* = 0.5), suggesting that these parameters (while related to mortality) are not suitable to predict persistent postprocedural transfusion need within this population (Figure 4).

## 5. Discussion

This study provides comprehensive real-world evidence on the clinical outcomes of patients undergoing left atrial appendage closure using the Watchman device, with a particular focus on bleeding risk, transfusion requirements, and long-term mortality. In an elderly, fragile, high-risk patient cohort characterized by advanced age, significant comorbidity burden (including high incidence of hypertension, diabetes mellitus, chronic kidney disease, previous cerebrovascular events, cardiovascular disease) and elevated thromboembolic and hemorrhagic risk scores, LAAC was performed with high technical success, consistent with findings from large multicenter cohorts such as the EWOLUTION trial (8). Procedural complications were infrequent and non-fatal, reaffirming the safety profile of LAAC reported in both clinical trials and registry studies [8,9].

During a mean follow-up of 1210 days (232 patient-years), one ischemic stroke and one transient ischemic attack occurred, yielding a combined cerebrovascular event rate of 0.86 per 100 patient-years. After successful appendage closure, residual cerebrovascular events predominantly reflect non-cardioembolic mechanisms rather than atrial fibrillation-related embolism. The incidence is comparable to or lower than rates reported in major LAAC trials and registries and might reflect the protective effect of postoperative applied antiplatelet therapy. These outcomes support the efficacy of the Watchman device in stroke prevention among patients with contraindications to long-term anticoagulation, aligning with data from large administrative databases and real-world studies [10,11].

The above-mentioned large-scale studies and registries comprehensively document postoperative thrombotic complications; however, analysis of bleeding events is limited and often lacks details. The first key finding of this study is the significant reduction of bleeding events following LAAC. Among patients at high bleeding risk, both the number of affected individuals and the total number of bleeding events declined significantly (51% and 35%, respectively) after cessation of anticoagulant therapy. These results are also in line with EWOLUTION study, which demonstrated a 46% reduction in expected bleeding rates compared to warfarin-treated populations [10]. We documented every pre- and postoperative bleeding event severity, origin, hospitalization and transfusion requirement. The number of TIMI and ISTH major bleeding and events with severe BARC categories were collectively reduced and intracranial hemorrhage was eliminated post-procedurally. When patients did not require preoperative transfusion, postoperative transfusion need was unlikely to develop, since only two patients receiving red blood cell supplements post-procedurally required no blood supplement before the procedure. Therefore, these data suggest that LAAC implantation provides a reliable and effective solution, preventing postoperative bleeding events. Our findings underscore the importance of carefully balancing complex antithrombotic strategies—including LAAC implantation—in frail patients with atrial fibrillation.

In our cohort, postprocedural management consisted uniformly of six-month dual antiplatelet therapy without routine short-term oral anticoagulation, reflecting the institutional practice and the manufacturer-recommended protocol. Given the highly selected, elderly population with extreme bleeding risk and frequent prior hemorrhagic events, the combination of oral anticoagulation and antiplatelet therapy was considered clinically unacceptable in many cases. While this strategy may limit direct comparison with studies applying short-term anticoagulation after LAAC, it ensured a homogeneous post-implant antithrombotic regimen and may have contributed to minimizing pharmacologically provoked bleeding events in this vulnerable population. It is important to note that substantial, residual bleeding risk persisted after LAAC, particularly during the first 6 months of DAPT. A short-term spike in transfusions is present immediately following LAAC, likely reflecting again the effect of periprocedural anticoagulant administration and transient dual antiplatelet therapy. Despite overall bleeding reduction, a subset of patients required hospital readmission and transfusions, indicating that bleeding risk is not completely abolished by LAAC and OAC omission in every individual. According to present guidelines, based on the balance of prothrombotic/bleeding factors, different postoperative antithrombotic regimens might be applied [12]. Recent registry data suggest that although all large number follow-up studies prove the effectivity of atrial closure in the prevention of ischemic stroke events from cardiac origin and device-related thrombotic complications, DAPT monotherapy was limited, with only 5% of patients receiving it in some cohorts [4]. In majority of the patients, oral anticoagulant therapy was continued after the intervention, unfortunately sustaining increased risk of postoperative bleeding events [4,12].

Because the majority of postprocedural bleeding events occurred during the early months after implantation, the intensity and duration of antithrombotic therapy might have contributed to the occurrence of transfusion-requiring bleeding. Emerging data and real-world registry experience suggest that less intensive approaches, including shorter DAPT or SAPT, may be considered in selected ultra-high-bleeding-risk patients, although the optimal regimen remains uncertain.

Our retrospective, non-randomized study design does not allow causal inference regarding the relationship between antithrombotic regimen and bleeding outcomes. Treatment selection was individualized and likely confounded by baseline bleeding risk. Further prospective studies are needed to determine whether regimen de-escalation could reduce early bleeding and transfusion requirements in this vulnerable population.

In our study, all patients were considered to have high-bleeding-risk status (HAS-BLED score median: 4); therefore, oral anticoagulants were replaced by dual antiplatelet therapy. It is important to note that we observed only one case (1.4%) of device-related thrombus without clinical consequence in a patient with Leiden mutation during follow-up. Without further precise data on coagulation and platelet parameters, decisions about antithrombotic regimen still remains an individual challenging clinical decision.

The other clinically most significant observation of this study is the robust association between postoperative transfusion need and mortality. Detailed data concerning clinical characteristics of individual bleeding events are often missing in large registries. Patients requiring red blood cell transfusions following LAAC had significantly higher overall and one-year mortality. Multivariate Cox regression suggests that need of pre- and postoperative transfusion was an independent predictor of death, with a six-fold increased risk.

To mitigate potential immortal-time bias and reverse causation, we performed landmark survival analyses at 45 and 90 days after LAAC implantation. The consistent separation of survival curves across transfusion groups further supports that postprocedural RBC transfusion is associated with substantially worse long-term outcomes, although it likely reflects an underlying high-risk clinical phenotype rather than a direct causal mechanism.

Interestingly, preprocedural bleeding and transfusions were associated with only a non-significant trend toward increased mortality, suggesting that recurrent severe bleeding episodes on DAPT, observed in the postprocedural period, are prognostically more relevant. This observation is in line with data showing that early post-LAAC complications, rather than baseline risk factors alone, determine outcomes [7]. In general, cessation of anticoagulant therapy prevents further bleeding events in most cases. However, in our cohort, 42% of the patients experienced bleeding events and 25% of patients required red blood cell transfusion after LAAC. Importantly, no deaths directly attributable to hemorrhagic shock were observed. Based on medical history, post-Watchman gastrointestinal bleeding has been identified as the most frequent hemorrhagic complication. This observation is consistent with the limited available literature data [13]. Identifying and managing the origin of the bleeding episode before LAAC implantation is crucial in reducing long-term mortality risk. Therefore, repeated gastrointestinal endoscopy and, when possible, interventions are highly recommended, stressing the complexity of managing bleeding complications in this population. Development of optimal medical therapy in these patients requires a multidisciplinary approach, involving not only cardiac interventionists but specialists in the field of internal medicine, gastroenterology and endoscopy.

While postprocedural RBC transfusion remained independently associated with mortality in multivariable models, this finding should be interpreted as associative rather than causal. Sustained postoperative transfusion events might be surrogate markers for frailty, ongoing bleeding tendency and increased vulnerability. Transfusion most likely represents a marker of residual bleeding susceptibility and overall clinical frailty, identifying a vulnerable patient subgroup with persistently elevated risk despite successful LAAC.

To improve patient selection and identify those at risk for postprocedural transfusion before the intervention, we explored potential demographic, clinical and echocardiographic predictors in the patients with postoperative transfusion needs. Atrial enlargement, impaired right ventricular function, and smoking and alcohol use emerged as significant predictors. Interestingly, commonly used risk stratification scores (CHA_2_DS_2_-VA, HAS-BLED) did not discriminate between patients with and without transfusion needs, although both were in the high range. This finding highlights the limitations of existing scoring systems, as they may not fully capture the procedural and bleeding-site-related risk factors in LAAC populations [9].

Besides the above-described robust effect of bleeding complications, similarly to previous studies, several clinical comorbidities such as diabetes and reduced renal function were also predictors of post-LAAC mortality in our study [14,15]. These parameters are independent of the intervention itself and rather reflect general properties of this multimorbid population. The relatively high overall mortality in our cohort likely reflects this advanced comorbidity burden and frailty of this elderly, highly selected population rather than a direct procedural consequence. In addition, our general population is characterized by particularly high baseline cardiovascular risk and malignancy burden, which may further contribute to adverse long-term outcomes. Although transfusion requirement might represent a marker of frailty, the significant reduction in transfusion need after LAAC suggests that withdrawal of oral anticoagulation may still meaningfully reduce bleeding burden.

The association between postprocedural transfusion and mortality observed in our study may partly reflect underlying frailty, multimorbidity, and progressive non-cardiovascular conditions such as malignancy or chronic kidney disease, rather than a direct causal relationship. Therefore, we now highlight again that transfusion might be interpreted as a potential marker of clinical vulnerability rather than a strictly causal determinant of mortality in this retrospective cohort.

## 6. Limitation

The primary limitation of this study is the relatively small sample size, which might limit its statistical power to detect modest associations. However, the small cohort size enabled detailed individual-level analysis of bleeding events, allowing us to assess each episode in terms of etiology, severity (based on standardized classification systems), hospitalization requirement, and transfusion burden. This provides valuable insight into the real-world clinical complexity of bleeding complications following LAAC which may be obscured in large registry datasets.

An additional limitation is the uniform use of six-month dual antiplatelet therapy without routine short-term oral anticoagulation following LAAC, reflecting the high bleeding risk of our study population and potentially limiting direct comparison with studies employing postprocedural anticoagulation. Similarly, in our cohort, all LAAC implantations were performed under transesophageal echocardiography (TEE) guidance. Although TEE remains the most widely used imaging modality for LAAC guidance, recent meta-analytic evidence suggests that ICE-guided procedures demonstrate comparable procedural success and safety outcomes [16]. Differences in imaging strategies across centers may therefore limit direct comparison with studies employing ICE-guided LAAC.

Finally, precise quantification of clinically relevant frailty was not feasible in this retrospective study, as most validated frailty indices require functional data consistently not available in the medical records.

## 7. Conclusions

Among a high-risk, comorbid population undergoing LAAC, we observed a significant reduction in bleeding events and transfusion requirements, with no thromboembolic deaths during follow-up. Furthermore, the need for postoperative red blood cell transfusion despite omission of oral anticoagulant therapy emerged as a strong, independent predictor of mortality. These findings underscore the critical prognostic impact of bleeding complications and emphasize the need for improved risk stratification, optimized antithrombotic regimens, and personalized postprocedural interdisciplinary management strategies to improve long-term outcomes following LAAC.

## Figures and Tables

**Table 1:** Clinical data and demographic parameters of the complete patient population and the two patient subgroups with recurrent stroke/thromboembolic event and high bleeding risk.

Demographic Data	Total Cohort (n = 70)	Patients with Recurrent Stroke/Thromboembolic Event (n = 8)	Patients with High Bleeding Risk (n = 62)
Characteristics			
Male/female, n (%)	37/33 (53/47)	3/5 (38/62)	34/28 (55/45)
Age, years	75.5 (68–80)	72.5 (65–77.5)	75.5 (68–80)
Comorbidities and risk factors			
Hypertension, n (%)	65 (93)	8 (100)	57 (92)
Diabetes mellitus, n (%)	24 (35)	3 (38)	21 (34)
Hyperlipidemia, n (%)	45 (64)	5 (63)	40 (65)
BMI, kg/m^2^ (M (IQR))	28 (24.6–30.5)	32 (21.3–35.2)	27.9 (24.7–30.1)
Chronic kidney disease, n (%)	43 (61)	4 (50)	39 (63)
Thyroid disease, n (%)	15 (21)	1 (13)	14 (23)
Previous epilepsy, n (%)	5 (7)	1 (13)	4 (7)
Smoking previously, n (%)	7 (10)	0 (0)	7 (11)
Current smoker, n (%)	10 (14)	1 (13)	9 (15)
Previous regular alcohol consumption, n (%)	2 (3)	0 (0)	2 (3)
Regular alcohol consumption, n (%)	13 (19)	2 (25)	11 (18)
Atherosclerotic cardiovascular disease			
CAD, n (%)	19 (27)	0 (0)	19 (31)
Previous carotid artery disease, n (%)	35 (50)	3 (38)	32 (52)
Previous sign. carotid artery disease, n (%)	1 (1)	0 (0)	1 (2)
Previous PAD, n (%)	10 (14)	1 (13)	9 (15)
Acute cardio/cerebrovascular events			
Previous AMI, n (%)	13 (19)	0 (0)	13 (21)
Previous TIA, n (%)	10 (14)	1 (13)	9 (15)
Previous stroke, n (%)	21 (30)	6 (75)	15 (24)
Previous other TE, n (%)	6 (9)	2 (25)	4 (7)
Previous hemorrhagic stroke, n (%)	11 (16)	2 (25)	9 (15)
Previous PCI, n (%)	11 (16)	0 (0)	11 (18)
Previous CABG, n (%)	7 (10)	0 (0)	7 (11)
Previous pacemaker implantation, n (%)	17 (24)	0 (0)	17 (27)
Risk stratification			
CHA_2_DS_2_-VASc Score	5 (4–6)	6 (5–6.5)	5 (4–6)
CHA_2_DS_2_-VA Score	4 (3–5)	5 (4.5–6)	4 (3–5)
HAS-BLED Score	4 (4–5)	4 (4–4.5)	4 (4–5)
Transthoracic echocardiogram			
Ejection fraction (%)	56 (46–60)	59 (50.5–65)	56 (42–60)
Left ventricular end-diastolic/ end-systolic diameter, mm	49 (46–55)/ 34 (30–42)	48 (46–52)/ 31 (28–37)	50 (46–55)/ 35 (29–42)
Interventricular septum/posterior wall diastolic thickness, mm	12 (10–13) 11 (10–13)	10.5 (10–12) 10 (10–11)	12 (10–13) 11 (10–13)
Right ventricular end-diastolic diameter, mm	37 (31–42)	33 (28–35)	38 (32–43) *
TAPSE, mm	19 (16–22)	22 (22–23.5)	19 (16–22)
Right atrium diameter, mm	58 (52–62)	51 (47.5–54)	60 (54–63) **
Left atrium diameter, mm	58 (53–64)	53 (49.5–55)	59 (54–64) ***

Cardiovascular risk factors and comorbidities were highly represented in the total population. However, in most of the demographic parameters, we found no statistically significant difference between patient subgroups (Mann–Whitney U and Fisher’s exact test for continuous and categoric parameters, respectively). Right ventricular end-diastolic diameter was larger in a subgroup of patients with high bleeding risk (* *p* = 0.048), as well as right and left atrium diameters (** *p* = 0.0047 and *** *p* = 0.029). Abbreviations: AMI: acute myocardial infarction, BMI: body mass index, CABG: coronary artery bypass grafting, CAD: coronary artery disease, IQR: interquartile range, M: median, PAD: peripheral artery disease, PCI: percutaneous coronary intervention, TAPSE: tricuspid annular plane systolic excursion, TE: thromboembolic event, TIA: transient ischemic attack.

**Table 2:** Antiplatelet and anticoagulant therapy of the total cohort and the two subgroups on admission and at discharge.

Medical Therapy	Total Cohort (n = 70)	Patients with Recurrent Stroke/Thromboembolic Event (n = 8)	Patients with High Bleeding Risk (n = 62)
Antiplatelet and anticoagulant therapy			
On admission (%)			
DAPT	7.1	0	8.1
ASA	15.7	12.5	16.1
Clopidogrel	20	12.5	21
VKA	10	25	8
DOACs	21.4	50	17.7
LMWH	42.8	12.5	46.8
At discharge (%)			
DAPT	88.6	50	93.5
ASA	92.9	87.5	93.5
Clopidogrel	95.7	62.5	100
VKA	2.9	12.5	1.6
DOACs	5.7	37.5	1.6
LMWH	0	0	0

Most frequently used anticoagulant was low-molecular-weight heparin on admission, while after the procedure dual antiplatelet therapy was applied in the majority of patients and LMWH was discontinued. One third of the patients with recurrent thromboembolic events remained on NOAC. Abbreviations: ASA: acetylsalicylic acid, DOACs: direct oral anticoagulants, LMWH: low-molecular-weight heparin, VKA: vitamin K antagonist.

**Table 3:** Cardiovascular endpoints and causes of death of total cohort and subgroups of patients with recurrent stroke/TE event and high bleeding risk.

Major Adverse Events and Mortality	Total Cohort (n = 70)	Patients with Recurrent Stroke/TE Event (n = 8)	Patients with High Bleeding Risk (n = 62)
Ischemic stroke/TIA, n (%)	1 (1.4)/1 (1.4)	0/0	1 (1.6)/1 (1.6)
Cardiovascular endpoints			
Coronary revascularization, n (%)	7 (10)	1 (12.5)	6 (9.7)
AMI, n (%)	1 (1.4)	0 (0)	1 (1.6)
Heart failure requiring hospitalization, n (%)	12 (17.1)	1 (12.5)	11 (17.7)
Pacemaker implantation, n (%)	7 (10)	0 (0)	7 (11.3)
New malignancy, n (%)	8 (11.4)	0/0	8 (12.9)
Mortality			
Number of deaths, n (%)	28 (40)	4 (50)	24 (38.7)
One-year mortality, n (%)	9 (12.9)	1 (12.5)	8 (12.9)
Cause of death			
Stroke, n (%)	0 (0)	0 (0)	0 (0)
Hemorrhagic shock, n (%)	0 (0)	0 (0)	0 (0)
Heart failure, n (%)	7 (25)	0 (0)	7 (29.1)
Infectious diseases, n (%)	9 (32.1)	2 (50)	7 (29.1)
Septicemia, n (%)	3 (10.7)	0 (0)	3 (12.5)
Infective endocarditis, n (%)	1 (3.6)	0 (0)	1 (4.2)
COVID-19 pneumonia, n (%)	4 (14.3)	1 (25)	3 (12.5)
Malignant neoplasms, n (%)	2 (7.1)	0 (0)	2 (8.3)
Asphyxiation, n (%)	2 (7.1)	1 (25)	1 (4.2)
Sudden death, n (%)	4 (14.3)	0 (0)	4 (16.7)
Unknown, n (%)	4 (14.3)	1 (25)	3 (12.5)

No statistical difference was found between the subgroups in respect to cause of death and cardiovascular endpoints.

**Table 4:** Clinical data of bleeding events, number of hospitalizations due to bleeding and applied red blood cell transfusions [units] before and after LAAC in a subgroup of patients with high bleeding risk.

	Preoperative	Postoperative
Frequency and severity of bleeding episodes		
No. of bleeding patients, n	61	30 *
No. of bleeding events, n	144	56
No. of bleeding events/patient, n/pt	2.4	1.9 *
No. of all hospital admissions (n)	n.a.	133
No. of hospitalizations due to bleeding (n)	110	34
No. of transfused patients (n)	33	19 *
No. of administered red blood cell supplements (n)	289	134
Average of administered red blood cell supplements/patients (n)	8.8	7.1 *
Cause of bleeding		
Gastrointestinal bleeding, n (%)	39 (63.9)	16 (53.3)
Hematuria, n (%)	4 (6.6)	1 (3.3)
Intracranial bleeding, n (%)	8 (13.1)	0 (0)
Epistaxis, n (%)	0 (0)	1 (3.3)
Hyphema, n (%)	1 (1.6)	1 (3.3)
Gastrointestinal bleeding and hematuria, n (%)	1 (1.6)	0 (0)
Gastrointestinal bleeding and epistaxis, n (%)	1 (1.6)	0 (0)
Gastrointestinal bleeding, hematuria and epistaxis, n (%)	0 (0)	1 (3.3)
Hematuria and epistaxis, n (%)	1 (1.6)	0 (0)
Intracranial bleeding and epistaxis, n (%)	1 (1.6)	0 (0)
Intracranial bleeding and hematuria, n (%)	1 (1.6)	0 (0)
Hematuria and scrotal bleeding, n (%)	0 (0)	1 (3.3)
Hemarthrosis, n (%)	0 (0)	1 (3.3)
Unknown, n (%)	4 (6.6)	5 (16.6)
Procedure-related bleeding	n.a.	3 (10)
Pericardial tamponade, n (%)	n.a.	1 (3.3)
Access site hematoma, n (%)	n.a.	2 (6.6)

In patients with high bleeding risk, LAAC implantation was associated with a significant reduction in bleeding burden (for paired cathegorical data, McNemar test, and for continuous data, Wilcoxon signed-rank test, *p* < 0.001, * indicates *p* < 0.05). Abbreviation: n.a.: not applicable.

**Table 5:** Demographics, clinical and echocardiography parameters of patients with and without red blood cell transfusion after the LAAC procedure. Out of the 62 successful procedures in patients with high bleeding risk, 19 patients required postoperative red blood cell transfusion.

	Patients with Postoperative Transfusion (n = 19)	Patients with No Postoperative Transfusion (n = 43)
Characteristics		
Male/female, n (%)	10/9 (52.6/47.4)	24/19 (55.8/44.2)
Age, years	73 (65–79)	76 (69–80)
Comorbidities and risk factors		
Hypertension, n (%)	17 (89.5)	40 (93)
Diabetes mellitus, n (%)	8 (42.1)	13 (30.2)
Hyperlipidemia, n (%)	12 (63.2)	28 (65.1)
BMI, kg/m^2^	27.7 (24.2–30.1)	28.1 (24.9–30.1)
Chronic kidney disease, n (%)	15 (78.9)	24 (55.8)
Thyroid disease, n (%)	5 (26.3)	9 (20.9)
Previous epilepsy, n (%)	2 (10.5)	2 (4.7)
Smoking previously, n (%)	3 (15.8)	2 (4.7)
Current smoker, n (%)	7 (36.8)	4 (9.3) *
Previous regular alcohol consumption, n (%)	1(5.3)	6 (14)
Regular alcohol consumption, n (%)	5 (26.3)	1 (2.3) *
Atherosclerotic cardiovascular disease		
CAD, n (%)	8 (42.1)	11 (25.6)
Previous carotid artery disease, n (%)	11 (57.9)	21 (48.3)
Previous sign. carotid artery disease, n (%)	0 (0)	1 (2.3)
Previous PAD, n (%)	2 (10.5)	7 (16.3)
Acute cardio/cerebrovascular events		
Previous AMI, n (%)	6 (31.6)	7 (16.3)
Previous TIA, n (%)	3 (15.8)	6 (14)
Previous stroke, n (%)	3 (15.8)	12 (27.9)
Previous thromboembolic event—other, n (%)	2 (10.5)	2 (4.7)
Previous hemorrhagic stroke, n (%)	1 (5.3)	8 (18.6)
Previous PCI, n (%)	3 (15.8)	8 (18.6)
Previous CABG, n (%)	4 (21.1)	3 (7)
Previous pacemaker implantation, n (%)	5 (26.3)	12 (27.9)
Risk stratification		
CHA_2_DS_2_-VASc Score	4 (4–6)	5 (4–6)
CHA_2_DS_2_-VA Score	4 (3–5)	4 (3–5)
HAS-BLED Score	4 (4–5)	5 (4–5)
Transthoracic echocardiogram		
Ejection fraction (%)	55 (44.5–58)	56 (42–61)
Left ventricular end-diastolic/ end-systolic diameter, mm	50 (47–61)/ 35 (26–48)	48 (46–55)/ 34 (30–40)
Interventricular septum/ Posterior wall diastolic thickness, mm	12.5 (12–13)/ 12 (11–13)	11 (10–13) */ 11 (10–13)
Right ventricular end-diastolic diameter, mm	42 (30–46)	37 (32–41)
TAPSE, mm	16 (14–22)	19 (16–22)
Right atrium diameter, mm	60 (57–70)	58 (50–62) *
Left atrium diameter, mm	64 (57–72)	58 (52–63) *
Preoperative bleeding		
Number of bleeding events, n	64	80
Number of patients, n	19	43
Bleeding event/patient, average	3.4 (+/−3.1)	1.9 (+/−1.0)
Preop. transfusion, n, total units	178	111 *
Preop. transfusion, n/patient units	9.9	2.6
Postoperative bleeding		
Postop bleeding event, total, n	40	16 *
Postop bleeding event/patient, n	2.1	0.4
Mortality data		
Death, n (%)	12 (63.2)	12 (27.9)
One-year mortality, n (%)	6 (31.6)	2 (4.6) *
Survived days in deceased patients, mean SD	874 ± 1037	1791 ± 1157

Subgroups were compared with Mann–Whitney U and Fisher’s exact test for continuous and categoric parameters, respectively. In most of the clinical parameters, we found no statistically significant difference. Prevalence of smoking, alcohol consumption and atrial diameters were higher in patients with postoperative transfusions (* *p* < 0.05). Abbreviations: AMI: acute myocardial infarction, BMI: body mass index, CABG: coronary artery bypass grafting, CAD: coronary artery disease, PAD: peripheral artery disease, PCI: percutaneous coronary intervention, TAPSE: tricuspid annular plane systolic excursion, TIA: transient ischemic attack.

## Data Availability

The data presented in this study are available on request from the corresponding author.

## Supplementary Materials

The following supporting information can be downloaded at: https://www.mdpi.com/article/10.3390/jcm15072626/s1, Table S1. Laboratory parameters of the complete cohort and the two patient subgroups with recurrent stroke/thromboembolic event and high bleeding risk; Table S2. Medical therapy of the total cohort and the two subgroups on admission and at discharge; Table S3. Clinical data and demographic parameters of survivor and deceased patient groups at the end of follow up; Table S4. Procedure-related complications of total cohort and subgroups of patients with recurrent stroke/TE event and high bleeding risk.

## Abbreviations

ACC	American College of Cardiology
ACCP	American College of Clinical Pharmacy
AF	atrial fibrillation
AHA	American Heart Association
AMI	acute myocardial injury
BARC	Bleeding Academic Research Consortium
CAD	coronary artery disease
DAPT	dual antiplatelet therapy
DOAC	direct oral anticoagulant
EESZT	National eHealth Infrastructure
ESC	European Society of Cardiology
HRS	Heart Rhythm Society
IQR	interquartile range
ISTH	International Society on Thrombosis and Hemostasis
LAA	left atrial appendage
LAAC	left atrial appendage closure
LAAO	left atrial appendage occlusion
MAE	major adverse events
OAC	oral anticoagulant
RBC	red blood cell
ROC	receiver operating characteristic
SAPT	single antiplatelet therapy
SEM	standard error of the mean
TIMI	Thrombolysis in Myocardial Infarction
TOE	transesophageal echocardiogram
TTE	transthoracic echocardiography
VKA	vitamin K antagonist
