# Supplementary material for: Recurring Bleeding Events Requiring Red Blood Cell Transfusion After Left Atrial Appendage Closure Are Associated with Increased Mortality
Source: J Clin Med. 2026 Mar 30;15(7):2626. doi: 10.3390/jcm15072626 (PMC13073375; doi:10.3390/jcm15072626)
Supplement: Supplementary file 1 [file jcm-15-02626-s001.zip › jcm-4187714-SI.pdf]

Supplement Table 1.

Laboratory parameters	Total cohort (n=70)	Patients with recurrent stroke/ thromboembolic event (n=8)	Patients with high bleeding risk (n=62)
Na (mmol/l)	140 (138-142)	140 (139-142)	140 (137-142)
K (mmol/l)	4.3 (4-4.6)	4.3 (4.2-4.5)	4.3 (4-4.7)
CN (mmol/l)	7.9 (6-11)	6.4 (5.7-6.9)	8.2 (6-12)
Creatinine (umol/l)	106.5 (82-138)	97 (83-106.5)	109.5 (82-143)
Red blood cell count (T/l)	4.2 (3.8-4.7)	4.4 (4.1-5.1)	4.2 (3.7-4.7)
Hemoglobin (g/l)			
on admission	122.5 (109-139)	126 (116.5- 148)	122 (107-137)
at discharge	111 (97-124)	114 (111-119)	111 (95-124)
Platelet count (G/l)			
on admission	217 (176-257)	211 (182.5-255.5)	217 (175-257)
at discharge	**182.5 (159.5-241.5)**	**180 (166-237)**	**185 (159-243)**
**Inflammatory markers**			
CRP (ml/l)			
on admission	2.75 (1.7-6)	2.1 (0.8-3.7)	2.8 (1.9-6.7)
at discharge	**7.9 (3.6-19.8)**	**6.3 (3.3-12.4)**	**7.9 (5-20.1)**
Leukocyte count (G/l)			
on admission	6.9 (5.6-7.8)	6.6 (5.4-7.4)	6.9 (5.5-7.9)
at discharge	7.4 (5.7-8.6)	7.8 (7.1-8.6)	7.4 (5.6-8.6)

Supplement Table 1. Laboratory parameters of the complete cohort and the two patient subgroups with recurrent stroke/thromboembolic event and high bleeding risk.

No statistical difference was found between the subgroups in respect to laboratory parameters. Minor elevation of leukocyte count and CRP was detected after the procedure. Slight reduction was detected at discharge in platelet number.

Continuous parameters provided as median (IQR).Marked but statistically not significant difference is highlighted with bold.

Abbreviations: CN: urea, CRP: C-reactive protein, Na: sodium, K: potassium.

Supplement Table 2.

Medical therapy	Total cohort (n=70)	Patients with recurrent stroke/ thromboembolic event (n=8)	Patients with high bleeding risk (n=62)
**Antiarrhythmic drugs**			
on admission (%)			
Beta blocker	82.9	62.5	85.5
Digoxin	15.7	12.5	16.1
Amiodarone	8.6	0	9.7
Propafenone	2.9	12.5	1.6
at discharge (%)			
Beta blocker	87.3	87.5	87.3
Digoxin	15.5	12.5	15.9
Amiodarone	9.9	0	11.1
Propafenone	2.8	12.5	1.6
**Other medical therapy**			
on admission (%)			
ACEi	40	50	38.7
ARB	30	12.5	32.3
ARNI	1.4	0	1.6
CCB	24.2	25	24.3
Nitrate	31.4	25	32.3
Statin	57.1	75	54.8
Alpha blocker	5.7	0	6.5
MRA	41.4	12.5	45.2
Centrally acting antihypertensives	5.6	0	6.5
Diuretics	67.1	25	72.6
OAD/Insulin	21.4/11.4	37.5/12.5	19.4/11.3
PPIs	74.3	75	74.2
at discharge (%)			
ACEi	42.9	50	41.9
ARB	30	12.5	32.3
ARNI	1.4	0	1.6
CCB	25.7	25	25.8
Nitrate	31.4	25	32.3
Statin	58.6	75	56.5
Alpha blocker	8.6	0	9.7
MRA	40	12.5	43.5
Centrally acting antihypertensives	4.3	0	4.8
Diuretics	65.7	25	71
OAD/Insulin	21.4/8.6	37.5/12.5	19.4/8.1
PPIs	98.6	100	98.4

Supplement Table 2. Medical therapy of the total cohort and the two subgroups on admission and at discharge.

No statistical difference was found between patient groups with recurrent stroke/thromboembolic event and high bleeding risk in respect to medical therapy on admission and at discharge.

Abbreviations: ACEi: angiotensin-converting-enzyme inhibitors, ARB: angiotensin receptor blocker, ARNI: angiotensin receptor/ neprilysin inhibitor, CCB: calcium channel blockers, MRA: mineralocorticoid receptor antagonist, OAD: oral antidiabetic drug, PPIs: proton pump inhibitors.

Supplement Table 3.

**Demographic data**	**Survivor (n=42)**	**Deceased (n=28)**
**Characteristic**		
Male/female, n (%)	24/18 (57.1/42.9)	13/15 (46.4/56.6)
Age, years	76 (69-80)	74 (68-80)
**Comorbidities and risk factors**		
Hypertension, n (%)	39 (92.9)	26 (92.9)
Diabetes mellitus, n (%)	9 (21.4)	**15 (53.6)**
Hyperlipidemia, n (%)	24 (57.1)	21 (75)
BMI, kg/m^2	26.4 (24.5-29.4)	**29.7 (27.4-32.6)**
Chronic kidney disease, n (%)	24 (57.1)	19 (67.9)
Thyroid disease, n (%)	9 (21.4)	9 (23.1)
Previous epilepsy, n (%)	2 (4.8)	3 (10.7)
Smoking previously, n (%)	3 (7.1)	**4 (14.3)**
Current smoker, n (%)	5 (11.9)	**5 (17.9)**
Previous regular alcohol consumption, n (%)	2 (4.8)	0 (0)
Regular alcohol consumption, n (%)	8 (19)	5 (17.9)
**Atherosclerotic cardiovascular disease**		
CAD, n (%)	9 (21.4)	**10 (35.7)**
Previous carotid artery disease, n (%)	20 (47.6)	15 (53.6)
Previous sign. carotid artery disease, n (%)	1 (2.4)	0 (0)
Previous PAD, n (%)	5 (11.9)	5 (17.9)
**Acute cardio/cerebrovascular events**		
Previous AMI, n (%)	6 (14.3)	**7 (25)**
Previous TIA, n (%)	5 (11.9)	5 (17.9)
Previous stroke, n (%)	11 (26.2)	**10 (35.7)**
Previous thromboembolic event - other, n (%)	4 (9.5)	2 (7.1)
Previous hemorrhagic stroke, n (%)	6 (14.3)	5 (17.9)
Previous PCI, n (%)	4 (9.5)	**7 (25)**
Previous CABG, n (%)	3 (7.1)	**4 (14.3)**
Previous pacemaker implantation, n (%)	8 (19)	**9 (32.1)**
**Risk stratification**		
CHA_2DS_2-VASc Score	4 (3-6)	**5.5 (4-7)***
CHA_2DS_2-VA Score	4 (3-5)	**5 (4-6)****
HAS-BLED Score	4 (4-5)	4 (4-5)

Transthoracic echocardiogram		
Ejection fraction (%)	55 (46-60)	58 (43-63)
Left ventricular end-diastolic / end-systolic diameter, mm	49.5 (46-55)/ 35 (32-42)	48 (46-57)/ 31 (26-41.5)
Interventricular septum/ Posterior wall diastolic thickness, mm	11 (10-13)/ 11 (10-13)	12 (11-13)/ 12 (10.5-13)
Right ventricular end-diastolic diameter, mm	38.5 (34-42)	**32 (29-41)***
TAPSE, mm	19 (16-22)	19 (15-22)
Right atrium diameter, mm	60 (51-63)	57 (53-60)
Left atrium diameter, mm	59 (53-65)	57.5 (53-62)

Supplement Table 3. Clinical data and demographic parameters of survivor and deceased patient groups at the end of follow up.

Significant difference was found between the survivor and deceased patient groups in respect to CHA_2DS_2-VASc score (*p=0.01), CHA_2DS_2-VA score (**p=0.013) and right ventricular end-diastolic diameter (***p=0.04) by Mann-Whitney U test. In respect to several demographic parameters (diabetes mellitus, BMI, smoking, coronary artery disease (CAD, PCI, CABG), previous cerebrovascular event, prior pacemaker implantation) the difference did not reach the statistical significance, although all parameters were highly represented among deceased patients.

Continuous parameters provided as median (IQR).Marked but statistically not significant difference is highlighted with bold.

Abbreviations: AMI: acute myocardial infarction, BMI: body mass index, CABG: coronary artery bypass grafting, CAD: coronary artery disease, PAD: peripheral artery disease, PCI: percutaneous coronary intervention, TAPSE: tricuspid annular plane systolic excursion, TIA: transient ischemic attack.

Supplement Table 4.

	**Total cohort (n=70)**	**Patients with recurrent stroke/TE event (n=8)**	**Patients with high bleeding risk (n=62)**
**Procedure information**			
Device size (mm)	27 (25.5-31)	25 (24-27)	30 (27-31)
**Procedure- related complication, n (%)**	7 (10)	1 (12.5)	6 (9.7)
Pericardial effusion, n (%)	1 (1.4)	1 (12.5)	0 (0)
Pericardial tamponade, n (%)	1 (1.4)	0 (0)	1 (1.6)
Major bleeding, n (%)	1 (1.4)	0 (0)	1 (1.6)
Vascular complication, n (%)	2 (1.4)	0 (0)	2 (3.2)
Esophageal injury, n (%)	1 (1.4)	0 (0)	1 (1.6)
Thrombus formation, n (%)	1 (1.4)	0 (0)	1 (1.6)
Ischemic stroke, n (%)	0 (0)	0 (0)	0 (0)
Procedure-related death, n (%)	0 (0)	0 (0)	0 (0)

Post-procedural complication during follow up	Total cohort (n=70)	Patients with recurrent stroke/TE event (n=8)	Patients with high bleeding risk (n=62)
Peri-device leak, n (%)	14 (20)	3 (37.5)	11 (17.7)
Significant peri-device leak, n (%)	0 (0)	0 (0)	0 (0)
Peri-device leak (mm)	2.75 (2-4)	2 (2-3)	3 (2-4)
Device-related thrombus, n (%)	1 (1.4)	0 (0)	1 (1.6)

Supplement Table 4. Procedure-related complications of total cohort and subgroups of patients with recurrent stroke/TE event and high bleeding risk.

No statistical difference was found between the subgroups in respect to acute and long-term procedure-related complications. Postprocedural transesophageal echocardiography was performed to exclude peri-device leak and device-related thrombus.
